# Level of Serum Enzymes and Electrocardiogram in Healthy Rabbits after Injection of ICD-85 as an Anticancer Agent

**DOI:** 10.7508/ibj.2015.04.003

**Published:** 2015-10

**Authors:** Abbas Zare Mirakabadi, Ali Sarzaeem

**Affiliations:** 1 *Dept. of Venomous Animals and Anitvenom Production, Razi Vaccine and Serum Research Institute, Karaj, Iran; *; 2 *Young Researchers Club, Karaj Branch, Islamic Azad University, Karaj, Iran*

**Keywords:** ICD-85, Electrocardiogram, Anticancer

## Abstract

**Background:**

Our previous *in vivo* studies confirmed that ICD-85, as an anticancer agent, was able to prevent further growth of breast tumors and expand the life expectancy of mice with breast cancer.

**Methods:**

Blood collection was carried out before, 1, 3, and 6 hours after ICD-85 injection. Sera were used to determinate the cardio and hepatic enzymes levels, including ALT, AST, LDH, CPK, and Ck-MB. Coagulation factors such as PT and PTT were also assayed. ECGs of all rabbits were recorded during the experiment.

**Results:**

ECG results showed that the injection of 50 and 100 µg/kg ICD-85 into healthy rabbits has no significant effect on heart function while the injection of 150 to 200 µg/kg ICD-85 caused ECG wave changes and mild bradycardia without toxic effects on heart. After ICD-85 injection (concentrations below 100 µg/kg), no significant increase was observed in liver and cardiac enzymes (ALT, AST, LDH, CPK, and CK-MB). However, the concentration of 150 µg/kg and above caused a rise in the enzymes. Comparison of the PT and PTT before and after ICD-85 injection showed no significant clotting time at any concentrations below 200 µg/kg.

**Conclusion:**

Based on the results obtained in the present study as well as our previous reports, ICD-85 at concentrations below 100 µg/kg seems to have no significant effect on the serum enzymes as indicators of hepatotoxicity and cardiotoxicity in healthy rabbits. However, to confirm this conclusion, more detailed surveys on heart and liver is needed to be carried out.

## INTRODUCTION

Our previous studies confirmed the anticancer effect of ICD-85 [1, 2]. Since some anticancer drugs may affect the cells of vital organs, such as heart, kidney, liver, bladder, lungs, and nervous system [3], oncologists continue to search for new anticancer drugs that are more potent and have fewer side effects [4, 5]. Cardiotoxicity and hepatotoxicity can be caused by many cytotoxic drugs; thus, liver function tests and cardiac biomarkers are groups of clinical biochemistry laboratory blood assays designed to give information about drug side effects [6, 7]. Commonly, the liver transaminases including ALT and AST are useful biomarkers of liver injury in the patients with some degrees of intact liver function [7]. In addition, LDH is an enzyme found in many body tissues such as liver and heart that its elevation may induce hepatic and cardiac damages [8]. The use of chemotherapeutic agents, radiation therapy, and molecular targeted therapies are all approaches that can injure the cardiovascular system, both at a central level by deteriorating the heart function and in the periphery by enhancing hemodynamic flow alteration and thrombotic events often latently present in oncology patients [6].

In medical toxicology, the electrocardiogram (ECG) and the measurement of cardiac biomarkers play an important role in the evaluation of drug cardiotoxicity [9]. CPK and CK-MB are cardiac markers used to evaluate the heart function and assist the diagnoses of acute myocardial infarction [10, 11]. PT and activated PTT are basic coagulation tests that determine the integrated actions of the majority coagulation factors in extrinsic and intrinsic pathways of blood coagulation cascade [12, 13].

Our previous *in vitro* studies showed that ICD-85, as an anticancer agent, had anti-proliferative effect and anti-angiogenesis activity on cancer cells through the induction of apoptosis without any significant effect on normal cells [14, 15]. Also, other clinical research confirmed that ICD-85 was able to prevent further growth of breast tumors and expand the life expectancy of mice with breast cancer [16]. Since cardiotoxicity, hepatotoxicity, and coagulation factors are important in using drugs, we prompted to evaluate the levels of rabbit serum enzymes after the injection of ICD-85 at different doses.

## MATERIAL AND METHODS

***Experimental animals. ***Healthy New Zealand white rabbits (n = 20) with an average weight of 1.5 ± 0.5 kg were selected. Animals were handled according to the rule of the Animal Research Committee. Prior to the experiment, rabbits were maintained in quarantine for at least 24 h. The environment was controlled at 18-22°C, and the animals had access to food and water. No animals were used in the experiment with any sign of ill health. The animals were divided into four groups. The rabbits were anesthetized with intra-muscular injection of ketamine and xylazine in a rate of 2:0.5 ml. The ICD-85 at four doses of 50, 100, 150, and 200 µg/kg of body weight was intravenously injected into groups 1, 2, 3 and 4, respectively.

***Blood sample collection. ***Blood was collected from the vein in the ear of the rabbits at 0, 1, 3, and 6 h after the injection of various doses of ICD-85. To separate the sera samples, the blood samples were centrifuged at 224 ×g at 4°C for 5 min. The samples were then stored at -20°C and analyzed within 48 h of collection.

***ICD-85 (venom-derived peptides). ***The ICD-85 was obtained from Razi Vaccine and Serum Research Institute (Karaj, Iran). The active fraction of ICD-85, which is a combination of three peptides with a molecular weight of about 10 to 30 kDa, were derived from the venom of the Iranian brown snake (*Agkistrodon halys*) and yellow scorpion (*Hemiscorpius lepturus*) [1, 2].

***Biochemistry parameters. ***Blood collection was carried out at 0, 1, 3, and 6 h. ICD-85 was injected into groups 1, 2, 3, and 4 with the concentrations of 50, 100, 150, and 200 µg/kg, respectively. The separated sera were used for the analysis of liver and cardiac biomarkers, including ALT, AST, LDH, CPK, and CK-MB, which were estimated by the colorimetric method [17]. PT and PTT levels were assayed by commercial kits. All kits were purchased from Pars Azmoon Company (Iran).

***Electrocardiography. ***ECGs of all rabbits before (as baseline) and after the ICD-85 injection were recorded during the experiment using an ECG recorder (ML-136 Animal Bio Amp, Australia). All observations were also carried out in limb lead II.

***Data analysis. ***The results were presented as mean ± SD. The results obtained before and after the injection of ICD-85 were compared using standard *t*-tests. *P-*values are indicated in the legends of all tables and figures. Statistical significant was accepted at a level of *P* < 0.05.

## RESULTS

***Clinical symptom after ICD-85 injection in rabbits. ***Injection of 50 and 100 µg/kg of ICD-85 to groups 1 and 2 showed no significant cytotoxic effects and shock at 1 and 3 h following ICD-85 injection. Therefore, no significant local signs were observed except a red circle of less than 0.3 cm around the injection site without inflammation during the experiment. When 150 and 200 µg/kg of ICD-85 was used in groups 3 and 4, heartbeat was decreased up to 10% and 30%, respectively. This change in HR returned to normal vital conditions after 24 h. There was also no significant effect on QRS or ST segment during the experiment.

***Effect of ICD-85 injection on hepatic and cardiac biomarkers in rabbits. ***Statistical analysis in groups 1 and 2 of animals showed that liver and heart biomarkers activity (ALT, AST, LDH, CPK, and CK-MB) did not alter significantly when animals received 50 and 100 µg/kg of ICD-85 (Tables 1 and 2). In groups 3 and 4, the injection of 150 and 200 µg/kg of ICD-85, respectively caused gradual increases in serum ALT, AST, LDH, CPK, and CK-MB. The rise was significant (*P* < 0.05) only in LDH at three hours after the injection as compared with the samples before the injection of ICD-85 (Tables 1 and 2).

***Coagulation activity in rabbit after the injection of ICD-85. ***PT and PTT activity in all groups of the animals did not change significantly following ICD-85 injection as compared with the samples before injection (Table 3).

**Fig. 1:**
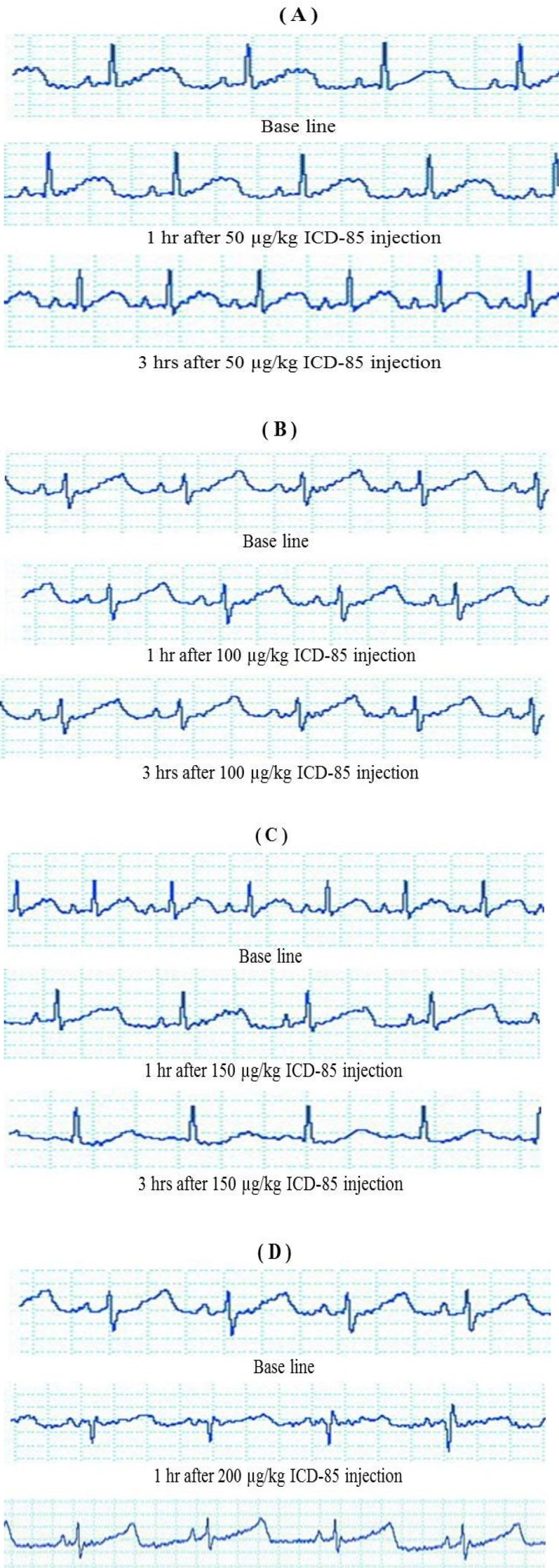
ECG recordings of rabbits after injection of various doses of ICD-85 at different times. The ECG of all rabbits before (as base line) and after the ICD-85 injection in each group. Injection of 50 µg/kg (A), 100 µg/kg (B), 150 µg/kg (C), 200 µg/kg (D) of ICD-85

***Electrocardiogram. ***ECG in all the animals after ICD-85 injection was compared with baseline (before injection) in each group. ECG of all animals in groups 1 and 2 before and after ICD-85 injection are shown in, QRS wave, and T-wave. However, a mild bradycardia was occurred three hours after the ICD-85 injection, and in this situation, the HR was decreased from the average of 240-228 beat/min, and no depression in the ST segments was observed (Fig 1A and 1B). When group 3 was injected with 150 µg/kg ICD-85, no significant change was seen in the QRScomplexes and ST segments. However, a mild decreased HR was observed from the average of 229 beat / min to 163 beat/min, indicative of a mild bradycardia (Fig. 1C). In addition, when rabbits in group 4 were injected with 200 µg/kg ICD-85, the repolarization time and prolongation of QT interval did not change in comparison with baseline at one hour. However, three hours after ICD-85 injection, the R-R interval was changed from the average of 0.60 second to 0.120 second without widening QRS complex when compared to the baseline. The HR was significantly decreased from the average of 234 beat/min to 128 beat/min (Fig. 1D).

**Table 1:** Comparison of ALT, AST, and LDH serum levels in groups 1, 2, 3, and 4 at different times following intravenous injection of various ICD-85 doses

**Group**	**ICD-85 doses (**µg/kg)	**ALT (U/L)**				**AST (U/L)**				**LDH (U/L)**		
		**Before injection**	**1 h after injection**	**3 h after injection**		**Before injection**	**1 h after injection**	**3 hrs after injection**		**Before injection**	**1 h after injection **	**3 h after injection**
1	50	6.72 ± 3.91	7.18 ± 2.43 NS	9.32 ± 2.17 NS		10.27 ± 6.56	11.77 ± 5.1 NS	17.14 ± 4.10 NS		95.11 ± 36.2	96.33 ± 42.11 NS	95.71 ± 34.26 NS
												
2	100	7.02 ± 2.33	7.12 ± 2.53 NS	7.87 ± 1.93 NS		9.67 ± 6.26	12.21 ± 5.10 NS	11.97 ± 5.58 NS		112.21 ± 41.31	110.33 ± 48.51 NS	115.61 ± 37.01 NS
												
3	150	6.34 ± 3.13	6.12 ± 3.1 NS	8.37 ± 2.91 NS		12.69 ± 5.02	13.19 ± 5.18 NS	15.40 ± 5.21 NS		108.72 ± 48.37	154.91 ± 51.38 NS	171.24 ± 49.17 NS
												
4	200	7.62 ± 3.56	13.21 ± 3.3 *P* < 0.05	18.02 ± 3.98 *P* < 0.01		10.38 ± 5.25	15.47 ± 5.54 NS	25.27 ± 5.31 P *P* < 0.01		112.01 ± 46.16	127.71 ± 56.26 NS	203.21 ± 48.33 *P* < 0.05

The values represent the mean ± SD (standard deviation). Male healthy New Zealand white rabbits (n = 20, 1.5 ± 0.5 kg/body weight) were assigned into four groups (five rabbits in each group) for this experiment. Obtained analysis of sera samples after ICD-85 injection was compared with results obtained before injection. NS, none significant

**Table 2:** Comparison of CPK and CK-MB serum levels in groups 1, 2, 3, and 4 at different times following intravenous injection of various ICD-85 doses

**Group**	**ICD-85 doses (**µg/kg)	**CPK (U/L)**				**CK-MB (U/L)**		
		**Before** ** injection**	**3 h after ** **injection**	**6 h after** ** Injection**		**Before** ** injection**	**3 h after** ** injection**	**6 h after** ** injection**
1	50	159.73 ± 52.7	171.14 ± 49.61 NS	166.21 ± 59.98 NS		191.41 ± 86.26	206.41 ± 113.26 NS	218.53 ± 106.15 NS
								
2	100	141.38 ± 73.09	152.25 ± 68.54 NS	140.13 ± 71.89 NS		201.11 ± 84.31	225.92 ± 81.35 NS	230.41 ± 126.14 NS
								
3	150	155.01 ± 49.98	189.13 ± 52.34 NS	197.73 ± 61.28 *P* < 0.05		196.41 ± 96.26	241.21 ± 82.16 NS	253.31 ± 90.76 *P* < 0.05
								
4	200	148.11 ± 59.02	181.62 ± 60.98 NS	225.29 ± 62.77 *P* < 0.05		193.21 ± 80.19	261.32 ± 91.06 NS	287.29 ± 113.24 *P* < 0.05

The values represent the mean ± SD (standard deviation). Male healthy New Zealand white rabbits (n = 20, 1.5 ± 0.5 kg/body weight) were assigned into four groups (five rabbits in each group) for this experiment. Obtained analysis of sera samples after ICD-85 injection was compared with sera analysis before injection. NS, none significant

## DISCUSSION

There is much evidence that natural anticancer agents (derived from the animal venoms and plant extracts) may have adverse effects on the body's vital organs such as hepatotoxicity (liver damage) and cardiotoxicity (heart damage) during chemotherapy [3, 6, 18]. Changes of biochemical values are generally observed in organ failure and damage [19]. Farag *et al*. [20] reported that the levels of cardiac and hepatic enzymes are important biomarkers of myocardial necrosis, and their abnormal values indicate the possible toxicity symptoms. In this study, we investigated the changes of serum enzyme levels after the injection of ICD-85 (venom-derived peptides) in rabbits.

The liver conjugates and excretes bilirubin, synthesizes ALT, stores AST and so, changes in serum levels of these analytes have become diagnostic tools and markers for assessing the liver function [21]. In the present work, the injection of ICD-85 up to 100 µg/kg had no significant changes in ALT and AST levels while ICD-85 injection at higher doses (200 µg/kg) caused a significant increase in these biomarkers levels in blood after three hours (Table 1). Researchers have reported that increases in serum ALT and AST levels are suggestive of hepatic damage and toxicity of drugs [21, 22]. Therefore, our results confirm that ICD-85 has no toxic effect on hepatic cells at concentrations less than 100 µg/kg.

Also, obtained results of LDH serum levels revealed that ICD-85 has no necrotic and toxic effects on various cells at concentrations below 100µg/kg. The LDH is a cytoplasmic enzyme retained by viable cells with intact plasma membranes, but it is released from necrotic cells with damaged membranes. On the other hand, necrotic activity in the liver causes a release of abnormal quantities of AST and ALT enzymes in the blood [21, 23, 24]. Therefore, the unchanged level of these serum enzymes reveals that probably the low dose (less than 100 µg/kg) of ICD-85 has no cytotoxic effect on liver cells.

Our previous *in vitro* studies of necrotic effect versus apoptotic nature of ICD-85 on cancer and normal cells confirm the results of LDH, ALT, and AST obtained from the present study [2, 14, 25]. We observed that liver biomarkers levels were significantly (*P* < 0.01) increased in the serum of animals after injection of 200 µg/kg ICD-85. Much evidence revealed that some natural and chemical compounds, including camptothecin as an anticancer drug [24], ethanol [26], cyanide [27], and carbon tetrachloride [28] are capable of increasing serum LDH, AST, and ALT activities, suggesting their hepatotoxic effects, after injection of their high doses into animals and human. Hence, we can conclude that the ICD-85 may have hepatotoxic effects at high doses.

**Table 3:** Comparison of coagulation factors in groups 1, 2, 3, and 4 at different times following intravenous injection of various ICD-85 doses

**Group**	**ICD-85 doses**	**PT (s)**				**PTT (s)**		
		**Before injection**	**1 h after** ** injection**	**3 h after** **injection**		**Before injection**	**1 h after** ** injection**	**3 h after** **injection**
1	50	13.6 ± 0.3	12.8 ± 0.4 NS	13 ± 0.2 NS		27.3 ± 1.3	28.4 ± 1.6 NS	27.8 ± 1.1 NS
2	100	11.5 ± 0.4	11.6 ± 0.5 NS	12.1 ± 0.4 NS		31.2 ± 1.2	30.8 ± 1.1 NS	29.5 ± 1.4 NS
3	150	12.4 ± 0.3	12.7 ± 0.2 NS	14 ± 0.7 NS		26.1 ± 1.7	25 ± 2.2 NS	27 ± 2.6 NS
4	200	11.8 ± 0.3	12 ± 0.7 NS	13 ± 1 NS		29.5 ± 1.4	27 ± 3.3 NS	26 ± 2.6 NS

The values represent the mean ± SD (standard deviation). Male healthy New Zealand white rabbits (n = 20, 1.5 ± 0.5 kg/body weight) were assigned into four groups (five rabbits in each group) for this experiment. Obtained analysis of sera samples after ICD-85 injection was compared with sera analysis before injection. NS, none significant

Some natural compounds (derived from the animal venoms) with impact on coagulation factors may prolong or shorten the blood clotting process [29]. On the other hand, in this study we measured PT and PTT time after ICD-85 injection in rabbits. Prothrombin (coagulation factor II) is a protein in the blood that is proteolytically cleaved to form thrombin in the coagulation cascade, which ultimately fibrin formation caused the blood to clot [30]. The PT and PTT are basic coagulation tests that determine the integrated actions of the majority coagulation factors in extrinsic and intrinsic pathways of blood coagulation cascade [12, 13]. Our results revealed that all doses of ICD-85 used in the present experiment have no effect on the PT and PTT time. Actually, there is evidence that the impact of antineoplastic drugs on the coagulation cascade following chemotherapy can basically damage to the intimae of the vessels [6]. In another study by our group, when the ICD-85 was injected into mice with breast tumor (*in vivo*), no bleeding at the site of injection was observed, indicating that ICD-85 prevents angiogenesis and has no effect on the blood coagulation [15, 16].

In the present study, we also measured the cardio biomarkers such as CPK and CK-MB. The result indicated that CPK and CK-MB levels begin to rise within about three to six hours after a heart attack and in the bloodstream, they return to normal levels within about 12 to 48 h after the heart attack [31]. In the current study, CPK and CK-MB serum levels were not significantly increased after the injection of low concentrations of ICD-85 (50 and 100 µg/kg). However, clinical examination of all the animals showed a decrease in HR. The CPK and CK-MB levels can be increased in the blood as a result of the heart cell damage and heart muscle characteristic of a myocardial infarction during heart attack [31]. The rise observed in the CK-MB and CPK with doses of 150 and 200 µg/kg following ICD-85 injection may be an indicative of toxic effect of ICD-85 at high doses for heart. Furthermore, our obtained results of AST support the CK-MB and CPK results, but it increases more and remains longer than CK-MB and CPK during hepatic failure or inflammation [32].

In the present work, we recorded the ECGs of the rabbits before and after ICD-85 injection at various times. ECG is a key examination for evaluating the effects of drugs on the cardiovascular system and is performed routinely in toxicity and pharmacology studies [33]. Here, ECG results in groups 1 and 2 showed that ICD-85 injection did not affect the heart function up to 100 µg/kg. However, in group 3, ECG revealed decreased T-wave and P-wave amplitudes at three hours following ICD-85 injection. Several researches have reported that many natural products and toxins have cardiovascular effects, which leads to ECG changes including QRS complex, T-wave and P-wave amplitude changes, bradycardia, tachycardia and short or prolong QT interval [34, 35]. Also, one hour after the injection of 200 µg/kg ICD-85 into the rabbits in group 4, we observed no change in repolarization time and prolonging QT interval in comparison with baseline. There is also a report that many drugs are known to produce acquired long QT in both therapeutic dose and overdose while injecting a high dose of ICD-85 produced no such changes [9].

However, acute or subacute cardiotoxicity is characterized by either the occurrence of abnormalities in ventricular repolarization and electrocardiographic QT interval changes as the QT interval is often prolonged in overdoses involving cardiotoxicity [9, 10]. On the other hand, in group 4, after three hours, the R-R interval became more than one second without widening, of course QRS complex in clinical examination decreased heartbeat up to 45%. There is evidence that bradycardia as a guiding symptom in toxicology is limited by the fact that many end-stage poisonings may present with severe illness including a slow HR, often with a wide QRS complex.

However, drugs that have a direct cardiotoxic effect and slow the HR as a central element of their mechanism of action can be expected to present classic toxidromes in overdose, with aggressive treatment strategies tailored based on mechanism of action [9]. Therefore, ICD-85 may have a mild toxic effect on heart function with reversible bradycardia at high doses above 100 µg/kg of body weight in rabbits. In conclusion, based on the results obtained in the present study as well as our previous reports, ICD-85 at concentrations below 100 µg/kg has no significant effect on the serum enzymes, as indicators of hepatotoxicity and cardiotoxicity in healthy rabbits. However to confirm this conclusion, more detailed surveys on heart and liver should be carried out.
